# Depletion of Tissue-Specific Ion Transporters Causes Differential Expression of PRL Targets in Response to Increased Levels of Endogenous PRL

**DOI:** 10.3389/fendo.2018.00683

**Published:** 2018-11-20

**Authors:** Tingting Shu, Yuqin Shu, Yanping Gao, Xia Jin, Jiangyan He, Gang Zhai, Zhan Yin

**Affiliations:** ^1^State Key Laboratory of Freshwater Ecology and Biotechnology, Institute of Hydrobiology, Chinese Academy of Sciences, Wuhan, China; ^2^College of Advanced Agricultural Sciences, University of Chinese Academy of Sciences, Beijing, China

**Keywords:** prolactin, ion transporter, gills/skin, pronephric duct, *slc12a10.2*, *slc12a3*

## Abstract

Prolactin (PRL) has been considered a key regulator of ion uptake in zebrafish. The genes *slc12a10.2* and *slc12a3*, which are Na^+^ and chloride Cl^−^ co-transporters, have been reported to be regulated by PRL in freshwater fish. The integrative network of PRL signaling dissected from the knockout of tissue-specific downstream PRL ion transporters remains poor. In the present study, zebrafish models with increased endogenous levels of PRL were generated through the knockout of *slc12a10.2* or *slc12a3*, and the developmental consequences were analyzed. The increased levels of pituitary PRL were observed in both *slc12a10.2*- and *slc12a3*-deficient fish. Unlike the *slc12a3*-deficient fish, which could survive to adulthood, the *slc12a10.2*-deficient fish began to die at 9 days post-fertilization (dpf) and did not survive beyond 17 dpf. This survival defect is a result of defective Cl^−^ uptake in this mutant, indicating that Slc12a10.2 plays an essential role in Cl^−^ uptake. Intriguingly, compared to the levels in control fish, no significant differences in the levels of Na^+^ in the body were observed in *slc12a10.2*- or *slc12a3*-deficient zebrafish. The upregulations of the PRL downstream transporters, *slc9a3.2, slc12a10.2*, and *atp1a1a.5* were observed in *slc12a3*-deficient fish in both the gills/skin and the pronephric duct. However, this type of response was not observed in the pronephric duct of *slc12a10.2*-deficient fish, except under Na^+^-deprived conditions. Our results show that PRL is susceptible to deficiencies in downstream ion transporters. Moreover, both the gills/skin and pronephric duct show differential expression of downstream PRL targets in response to increased levels of pituitary PRL caused by the depletion of tissue-specific ion transporters.

## Introduction

The endocrine system plays a central role in the homeostatic regulation of the salt and water balance in vertebrates (1). Several key neuroendocrine factors were identified as regulators of epithelial ion movement, including cortisol, catecholamines (adrenaline and noradrenaline), parathyroid hormone, and prolactin (PRL) (2). In mammals, PRL regulates salt and water balance by increasing intestinal water and salt absorption and reducing renal Na^+^ and K^+^ excretion through modulating Na^+^-K^+^ ATPase activity (3, 4). In fish, PRL has long been considered a key hormone for freshwater adaptation by preventing both the loss of ions and the uptake of water (5–10). The role of PRL in fish osmoregulation was first demonstrated in the 1950s by studies on killifish (*Fundulus heteroclitus*), as *F. heteroclitus* were unable to survive in freshwater following hypophysectomy; however, PRL treatment allowed hypophysectomized killifish to survive (8). This spawned an intensive investigation into the osmoregulatory role of PRL in a series of euryhaline species. PRL promotes the conservation of ions and secretion of water by acting on the gills/skin, pronephric duct, gut, and urinary bladder (7). Correspondingly, the highest *prl* receptor expression levels were observed in the primary osmoregulatory organs (kidney, gills, and intestine) (11–15). In zebrafish, PRL receptors (PRLRs), PRLRa and PRLRb, have been observed in the gills and pronephric duct, respectively. It has been demonstrated that PRL binds to PRLRs that are present in osmoregulatory organs (16–18). The multiple forms of PRL receptor family have been retained following genome duplication events (19), and have distinct expression patterns and capacities to activate intracellular signaling pathways (20–22). These results imply that PRL signaling is complicated and may be differentially regulated in disparate osmoregulatory organs.

The genetic understanding of the mechanisms through which PRL regulates ion transporters has only recently been investigated. Breves et al. demonstrated that *slc12a10.2* represents a conserved transcriptional target of PRL in fishes, as pituitary-derived PRL regulates *slc12a10.2* expression in ionocytes of the Mozambique tilapia (23). Our previous study also demonstrated that PRL is a positive regulator of ion absorption and reabsorption by maintaining the transcription of several transporters in the gill/skin and pronephric duct in zebrafish (17). The expression levels of certain key Na^+^/Cl^−^ co-transporters in the gills (*slc12a10.2*), as well as, Na^+^/K^+^-ATPase subunits (*atp1a1a.5*), Na^+^/H^+^ exchangers (*slc9a3.2*) and Na^+^/Cl^−^ transporters (*slc12a3*) in the pronephros in *prl*-deficient larvae were downregulated at 5 days post-fertilization (dpf), which caused defects in the uptake of Na^+^/K^+^/Cl^−^ in *prl*-deficient zebrafish (17). Collectively, the previous studies have demonstrated that the transcriptional targets of PRL include *slc12a10.2* in gills, and *slc12a3, slc9a3.2*, and *atp1a1a.5* in pronephric duct. Although great progress has been made in previous studies supporting the hypothesis that PRL regulates the transcription of different ionocyte transporters, the regulatory network of PRL signaling has not been extensively investigated because the manipulation of ion transporters downstream of PRL remains poor.

Zebrafish has been recognized as a model organism for studying osmoregulation (24), as both Transcription Activator-Like Effector Nuclease (TALEN) and Clustered Regularly Interspaced Short Palindromic Repeats/CRISPR-associated system (CRISPR/Cas9) have been successfully adopted in zebrafish to knockout functional genes (25–27). Apart from the effective knockout strategy, zebrafish also possess several advantages as an experimental organism in osmoregulation research, including the applicability of molecular tools, ease of *in vivo* cellular observation, and rapid embryonic development (28). In particular, zebrafish possess specialized ionocytes, principally located in the gills of adults and in the skin of embryos and larvae, which facilitate active absorption of electrolytes from the medium (29). In freshwater fishes, the gills and pronephric duct are the most important organs responsible for ionoregulation. The gill and pronephric duct are involved in ion regulation owing to the presence of numerous ion channels, pumps, or exchangers (30). In a hypotonic freshwater environment, teleosts have to actively absorb ions from the water mainly via the gills and excrete large amounts of urine via the pronephric ducts in order to maintain the homeostasis of their body fluids (31, 32). In the zebrafish pronephric duct, ions are reabsorbed through active transcellular and passive paracellular transport processes. To eliminate the excess water gained from the environment, freshwater fish reabsorb approximately 95% of the NaCl that enters the glomerular filtrate and then produce diluted, hypoosmotic urine (30, 33, 34). Upon the coordinated corporation of the gills and pronephric duct, zebrafish have a high capacity for Na^+^/Cl^−^ uptake, even living in hypoosmotic waters (35).

In zebrafish, four members of the *slc12a* family were identified: *slc12a3, slc12a10.1, slc12a10.2*, and *slc12a10.3*. Among the four members, *slc12a3* is the ortholog to the mammalian *slc12a3* (also known as *ncc*), whereas *slc12a10.2* was recognized as *ncc2b* or *ncc-like* (36). During the larval stage, transcriptional abundance of *slc12a10.2* was observed in the gill region and in the skin of the yolk sac, in addition to the observed transcriptional abundance of *slc12a3* in the pronephric duct (17). In the present study, knockout of *slc12a10.2* and *slc12a3* was performed. Our results differ from previous findings obtained from the manipulation of the pituitary *prl* (5, 17). The developmental consequences of *slc12a10.2*- or *slc12a3*-deficent fish were analyzed. We found that increased levels of pituitary PRL was observed in both the *slc12a10.2*- and *slc12a3*-deficient fish, suggesting that the expression of PRL is susceptible to deficiencies in downstream ion transporters. We demonstrated that depletion of *slc12a10.2* results in lethality, which is caused by defective Cl^−^ uptake, whereas the *slc12a3*-deficient fish survived healthily into adulthood without impairments in the levels of Na^+^ and Cl^−^. We also observed an upregulation of the PRL target transporters, *slc9a3.2, slc12a10.2*, and *atp1a1a.5*, in the pronephric duct in *slc12a3*-deficient fish; however, upregulation of these genes were not observed in *slc12a10.2*-deficient fish, unless under Na^+^-deprived conditions. The integrative comparisons of the developmental consequences of *slc12a10.2*- and *slc12a3*-deficient fish demonstrate that the gills/skin and pronephric duct show differential responses of PRL transcriptional targets in response to the increased levels of PRL caused by the depletion of tissue-specific ion transporters.

## Materials and methods

### Zebrafish maintenance

All zebrafish (AB strain) were maintained in a circulated water system with a 14-h light and 10-h dark cycle at 28.5°C and fed newly hatched brine shrimp (*Artemia salina*). The developmental stages of zebrafish embryos were characterized as described previously (37). All fish experiments were conducted in accordance with the Guiding Principles for the Care and Use of Laboratory Animals and were approved by the Institute of Hydrobiology, Chinese Academy of Sciences (Approval ID: IHB2013724).

### Establishment of *slc12a10.2* and *slc12a3* knockout lines

The *slc12a10.2* and *slc12a3* knockout lines of zebrafish were generated via CRISPR/Cas9 technology. Guide RNA (gRNA), generated against the *slc12a10.2* or *slc12a3* target sequence in the first exon, was designed using an online software tool (http://zifit.partners.org/ZiFiT) (38). gRNA was synthesized from the PCR products of the pMD19T-gRNA plasmid using primers containing the target sequence and nominated sequence for the plasmid. PCR products were directly used for gRNA synthesis using the TranscriptAid T7 High Yield Transcription Kit following the manufacturer's constructions (K0441, Thermo Fisher Scientific, Waltham, MA, USA). Cas9 mRNA was synthesized from the pXT7-Cas9 plasmid using the T7 mMESSAGE mMACHINE mRNA Transcription Synthesis Kit (AM1344, Thermo Fisher Scientific) and purified using RNeasy Mini Kit (#74106, Qiagen, Hilden, Germany). The Cas9 mRNA and gRNA for *slc12a10.2* or *slc12a3* were combined for injection immediately before use (27). For mutation examination, the PCR products from the genomes of fish were run in PAGE gel after denaturation and annealing. The PAGE gel was stained with ethidium bromide for 15 min before examination. The homoduplexes and heteroduplexes were separated with heteroduplexes being slower in mobility (39). The primers for mutation examination are listed in Table 1. The heterozygous males and females in the F1 population were crossed to generate an F2 population that contained *slc12a10.2* or *slc12a3* homozygotes.

**Table 1:** Primers used in this study.

**Gene**	**Forward primer (5′-3′)**	**Reverse primer (5′-3′)**	**Product size (bp)**
**FOR GENOTYPING**			
*slc12a10.2*	GAGACTGAAAAGCTTGACGATG	CGTGTCTTTGGCGTAAAAGTC	211
*slc12a3*	TCAGGATGACCCCTCTCCAC	AAGTGGGACCCTACAAGCAT	371
**FOR qPCR ANALYSIS**			
*atp1a1a.5*	GGTTCTGTGCCTTCCCATATATG	CCTAAACGGGTGTTCATTTTG	189
*slc12a3*	GCTGGGGAAGATGCAAAGTG	CTAATGTGTTCAGACTGCGGAGC	163
*slc9a3.2*	GCGAAACCCACCCTGGCAAAC	GGCGAAGGAGTCTGTGGAGCG	148
*slc12a10.2*	GAACTCCCTCTACGCTGCTC	GCGGGACAGTTGATACCGAT	117
*prl*	ATCCTTATGCCACACGTCATCT	AACAGCAGAGACCGAGCCAA	102
*prlra*	AGGCAGTTCAATGCAGCACGA	GCACAGCGGCGGAAATCCTCAT	122
*prlrb*	GACTGGATCAAGCGGTGGAC	AGGCCACCACGGTAATGTTG	170
*β-actin*	CGGAATATCATCTGCTTGTA	CATCATCTCCAGCGAATC	140

### Whole mount *in situ* hybridization (WISH)

We performed WISH as described previously (40). cDNA of the following genes were used as anti-sense digoxigenin-labeled probes in our study: *prl, slc12a10.2, slc12a3, slc9a3.2*, and *atp1a1a.5*.

### Sodium influx analysis

The sodium uptake by gill ionocytes was visualized using sodium green staining (S6901, Thermo Fisher Scientific) as previously described (17). Live larvae at 6 dpf were incubated in 10 μM sodium green in egg water containing 0.1% dimethyl sulfoxide (DMSO) for 1 h at 37°C. The larvae were anesthetized for photography (NOL-LSM 710 microscope, Carl Zeiss, Oberkochen, Germany).

### Whole body ion concentration measurement

Ion measurement of the whole body was performed as previously described (17). Briefly, zebrafish were rinsed with double-deionized water and dried out at 56°C for 6 h. Then 100 μL nitric acid (71%) was added to the samples for digestion at 65°C overnight. For Na^+^, K^+^, Ca^2+^, and Mg^2+^ measurements, the digested solution was diluted with double-deionized water to a volume of 5 mL and measured using inductively coupled plasma-optical emission spectroscopy (ICP-OES, PerkinElmer-OPTIMA 8000DV, Waltham, MA, USA). For Cl^−^ measurements, zebrafish were sonicated in 0.5 mL double-deionized water for 10 min until homogeneous, and the supernatant was collected after centrifuging at 14,000 rpm for 10 min. The Cl^−^ concentrations of the supernatants were measured using a Chloride Colorimetric Assay Kit (K530-100, Biovision, San Francisco, CA, USA) according to the manufacturer's instructions.

### The rescue experiment with brackish water and artificial water with different compounds

In the rescue experiment, 50 larvae of the F2 population were maintained in system water with sea salt (5 g sea salt added to 1 L system water), Mix 1 (3.336 g NaCl, 0.113 g KCl, 0.311 g CaCl_2_, and 1.378 g MgCl_2_ to 1 L system water), Mix 2 (4.054 g Na_2_SO_4_, 0.132 g K_2_SO_4_, 0.364 g CaSO_4_, and 0.816 g MgSO_4_ to 1 L system water), or Mix 3 (0.113 g KCl, 4.500 g CaCl_2_, and 1.378 g MgCl_2_ to 1 L system water). The survival ratio was recorded for 20 days.

### The hypo-Na^+^ mediated Na^+^ uptake stress with different concentrations of Na^+^ in the artificial rearing medium

Twenty control or *slc12a10.2*-deficient larvae from the F2 population were maintained in artificial water with ultrahigh Na^+^ concentration (3.336 g NaCl, 0.113 g KCl, 1.378 g MgCl_2_, and 0.311 g CaCl_2_ to 1 L purified water), high Na^+^ concentration (2.502 g NaCl, 0.113 g KCl, 1.378 g MgCl_2_, and 1.360 g CaCl_2_ to 1 L purified water), medium Na^+^ concentration (1.668 g NaCl, 0.113 g KCl, 1.378 g MgCl_2_, and 2.409 g CaCl_2_ to 1 L purified water), low Na^+^ concentration (0.834 g NaCl, 0.113 g KCl, 1.378 g MgCl_2_, and 3.458 g CaCl_2_ to 1 L purified water), or 0-Na^+^ concentration (0.113 g KCl, 1.378 g MgCl_2_, and 4.507 g CaCl_2_ to 1 L purified water). At 14 dpf, the control or *slc12a10.2*-deficient larvae reared at the above medium were harvested for RNA extraction and quantitative real-time PCR (qPCR) (see below).

### RNA extraction and qPCR

Total RNA was extracted using TRIzol reagent, and cDNA was synthesized using an oligo (dT) 18 primer and RevertAid First-strand cDNA Synthesis Kit (K1622, Thermo Scientific, Waltham, MA, USA) according to the manufacturer's instructions. qPCR primers for *atp1a1a.5, slc12a3, slc9a3.2, slc12a10.2, prl, prlra, prlrb*, and β*-actin* were designed using the National Center for Biotechnology Information (NCBI) primer BLAST service, and are listed in Table 1. All mRNA levels were calculated as fold expression relative to the housekeeping gene, β*-actin*. Each sample was run in triplicate, and the results were expressed according to the method described previously (41). The primers for qPCR were validated by DNA sequencing and agarose gel electrophoresis of PCR products.

### Statistical analysis

Detailed information regarding the number of zebrafish used per experiment is provided in each individual experiment and the corresponding figure. All analyses were performed with the GraphPad Prism 5.0 software program, and the differences were assessed using the Student's *t*-test. The results were expressed as the mean ± SD. For all statistical comparisons, a *P* < 0.05 was used to indicate a statistically significant difference.

## Results

### Cas9-mediated knockout of *slc12a10.2*

**Figure 1:**
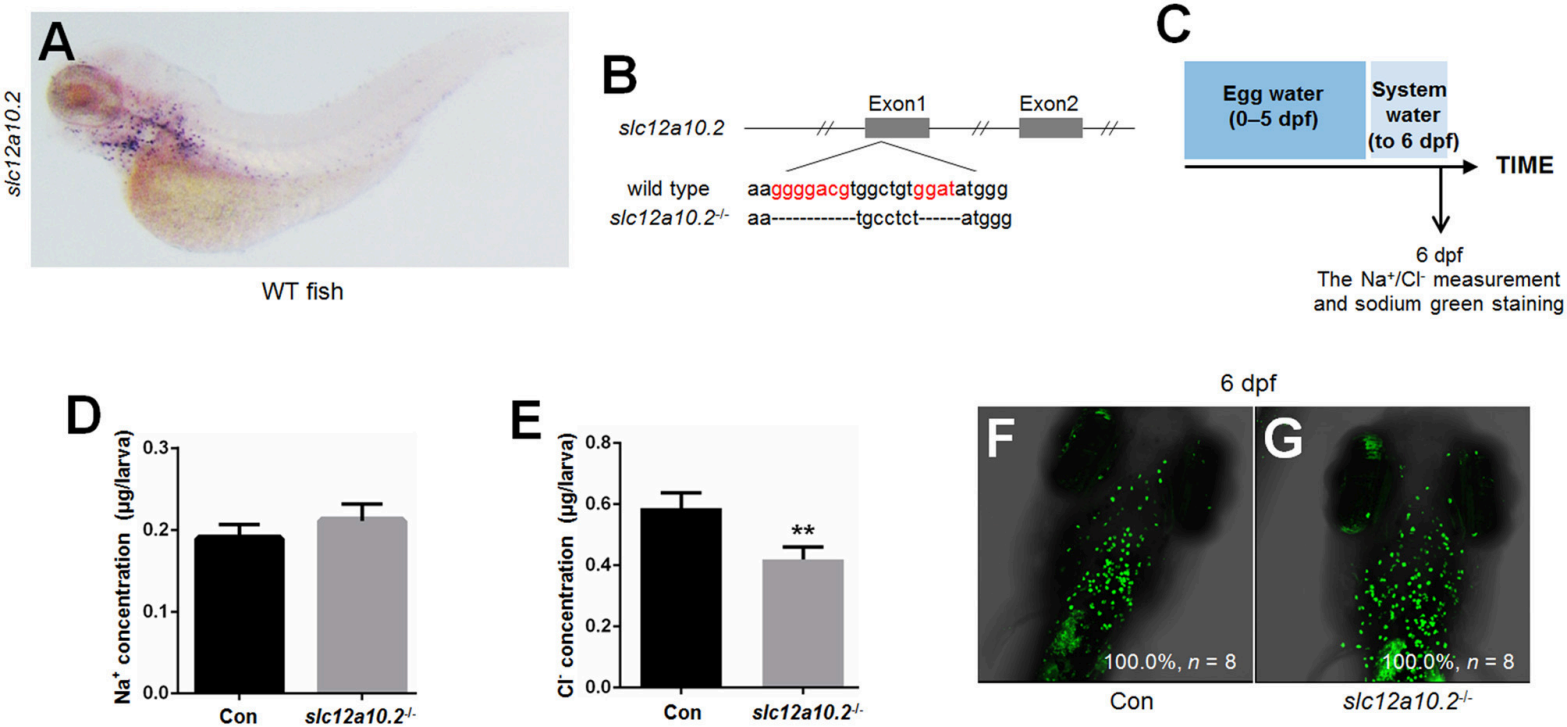
Generation of *slc12a10.2*-deficient zebrafish. **(A)** WISH analysis using the *slc12a10.2* probe in wild-type larvae at 4 dpf. **(B)** Targeted depletion of the *slc12a10.2* gene. The CRISPR/Cas9 target site is located at exon 1. A genotype with an 11-bp deletion was used to establish the *slc12a10.2* knockout line (highlighted in red). **(C)** Schematic diagram of the rearing timeline. Larvae were reared in egg water from 0 to 5 dpf and in system water after 5 dpf. The fish at 6 dpf were harvested for Na^+^ and Cl^−^ measurements. **(D,E)** Na^+^ and Cl^−^ concentrations were measured in control and *slc12a10.2*-deficient larvae at 6 dpf. ^**^P < 0.01. **(F,G)** Sodium green staining in the gills of control and *slc12a10.2*-deficient larvae at 6 dpf (ventral views).

WISH staining using a probe against *slc12a10.2* demonstrated that *slc12a10.2* is expressed in the gills/skin at 4 dpf (Figure 1A). A mutant line was obtained with an 11 base pair (bp) deletion in the first exon of *slc12a10.2* (Figure 1B). To evaluate the effects of *slc12a10.2*-deficiency on the Na^+^ and Cl^−^ concentrations in the entire zebrafish body, we measured the Na^+^ and Cl^−^ concentrations in *slc12a10.2*-deficient fish at 6 dpf. We observed unaffected Na^+^ concentration in the *slc12a10.2*-deficient fish (Figures 1C,D). This result demonstrates that the Na^+^ absorption is normally maintained in *slc12a10.2-*deficient fish. However, we did observe a significantly decreased Cl^−^ concentration in the *slc12a10.2*-deficient fish, suggesting an essential role of Slc12a10.2 in maintaining Cl^−^ levels within the body (Figure 1E). Sodium staining demonstrates that the *slc12a10.2*-deficient larvae at 6 dpf have normal Na^+^ uptake in gills (Figures 1F,G).

### Depletion of *slc12a10.2* resulted in lethality in freshwater

**Figure 2:**
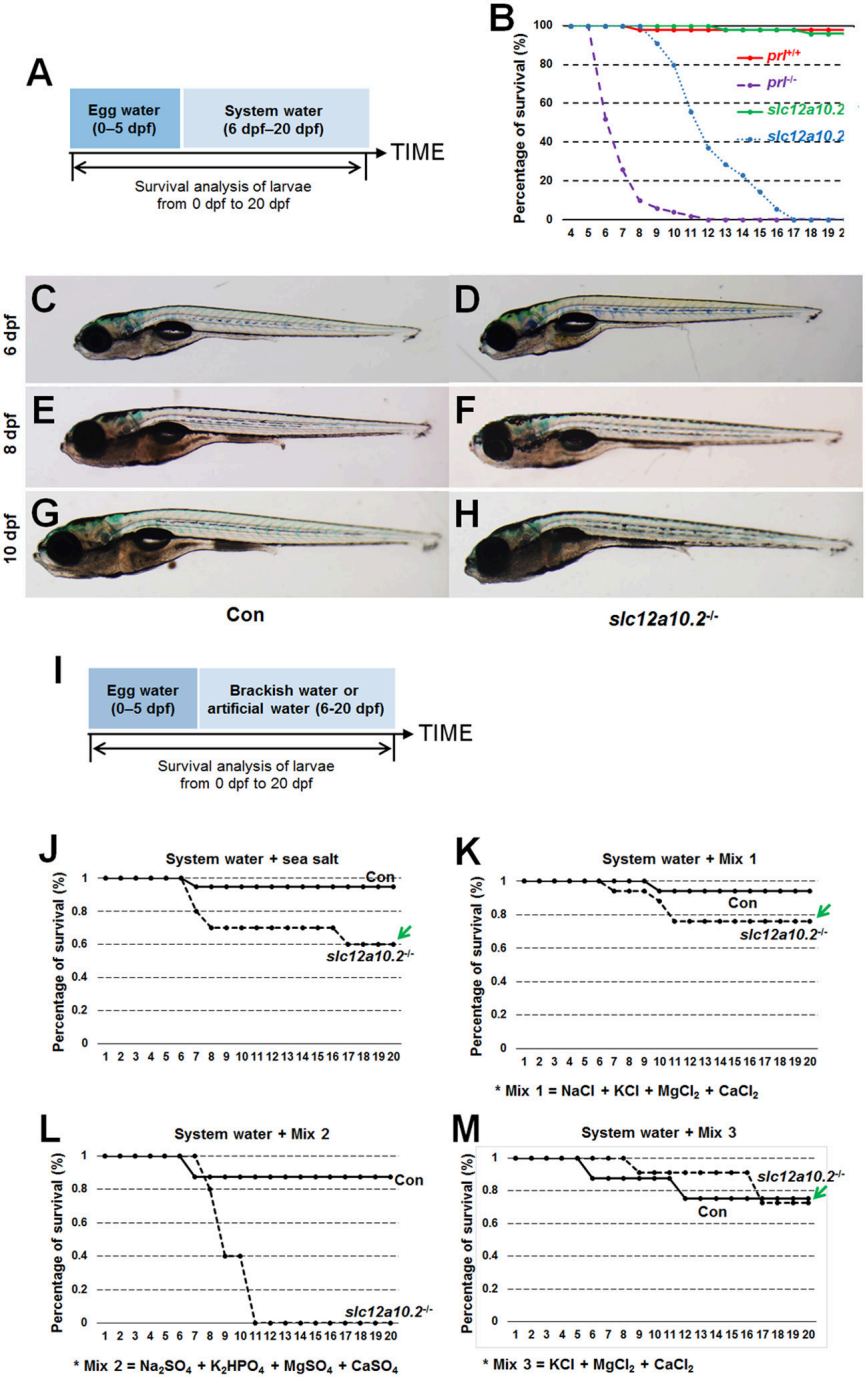
The lethality of the *slc12a10.2*-deficient zebrafish is rescued by exogenous Cl^−^ supplement. **(A)** Schematic diagram of the rearing timeline. Larvae were reared in egg water from 0 to 5 dpf, and in system water after 5 dpf. The survival ratio of fish reared under this condition was analyzed until 20 dpf. **(B)** Comparison of the survival ratios of *prl*^+/+^, *prl*^−/−^, *slc12a10.2*^+/+^, and *slc12a10.2*^−/−^ larvae (*n* = 25 for each genotype). **(C–H)** The general morphological observations of control larvae and *slc12a10.2*-deficient larvae at 6, 8, and 10 dpf. **(C,E,G)** Control larvae. **(D,F,H)** *slc12a10.2*-deficient larvae. **(I)** Schematic diagram of the rearing timeline. Larvae were reared in egg water from 0 to 5 dpf and in brackish or artificial water after 5 dpf. The survival ratios of fish reared under different conditions were analyzed until 20 dpf. **(J–M)** The survival ratios of control and *slc12a10.2*-deficient larvae in system water + sea salt, system water + Mix 1, system water + Mix 2, and system water + Mix 3.

When *slc12a10.2*-deficient larvae were reared under the standard conditions (egg water for the first 5 days and system water for the following 15 days), lethality occurred from 9 to 17 dpf (Figures 2A,B). The lethality of the *slc12a10.2*-deficient larvae occurred later than the *prl*-deficient larvae, as the lethality of the *prl*-deficient larvae occurred from 6 to 11 dpf (17) (Figure 2B). These results suggest that, compared to *prl*-deficient larvae, a more suitable ion concentration in the *slc12a10.2*-deficient zebrafish may be the reason for the delayed lethality in these fish. Based on our morphological observations of the early developmental processes of the fish at 6 and 8 dpf, no obvious defects could be found in the *slc12a10.2*-deficient fish (Figures 2C–F). However, significant body curvature and shrink defects were observed in the majority of the *slc12a10.2*-deficient fish at 10 dpf, and the defective conditions of these larvae continuously worsened over time (Figures 2G,H).

Our further analysis showed that the *slc12a10.2*-deficient zebrafish were able to survive healthily in sea salt-supplemented system water (Figures 2I,J). By providing additional Na^+^, K^+^, Mg^2+^, Ca^2+^, and Cl^−^ in the artificial water, the *slc12a10.2*-deficient zebrafish could survive healthily (Figure 2K). The rescue of lethality was observed when Na^+^ was depleted in the artificial water; however, the rescue failed when Cl^−^ in the artificial water was replaced with ${\text{SO}}_{4}^{2-}$ (Figures 2L,M). These results demonstrate that the *slc12a10.2*-deficient fish died owing to the defective uptake of Cl^−^, not Na^+^, clearly indicating an essential role of Slc12a10.2 in Cl^−^ uptake.

### The depletion of *slc12a10.2* upregulates PRL rather than PRL transcriptional targets in the pronephric duct

**Figure 3:**
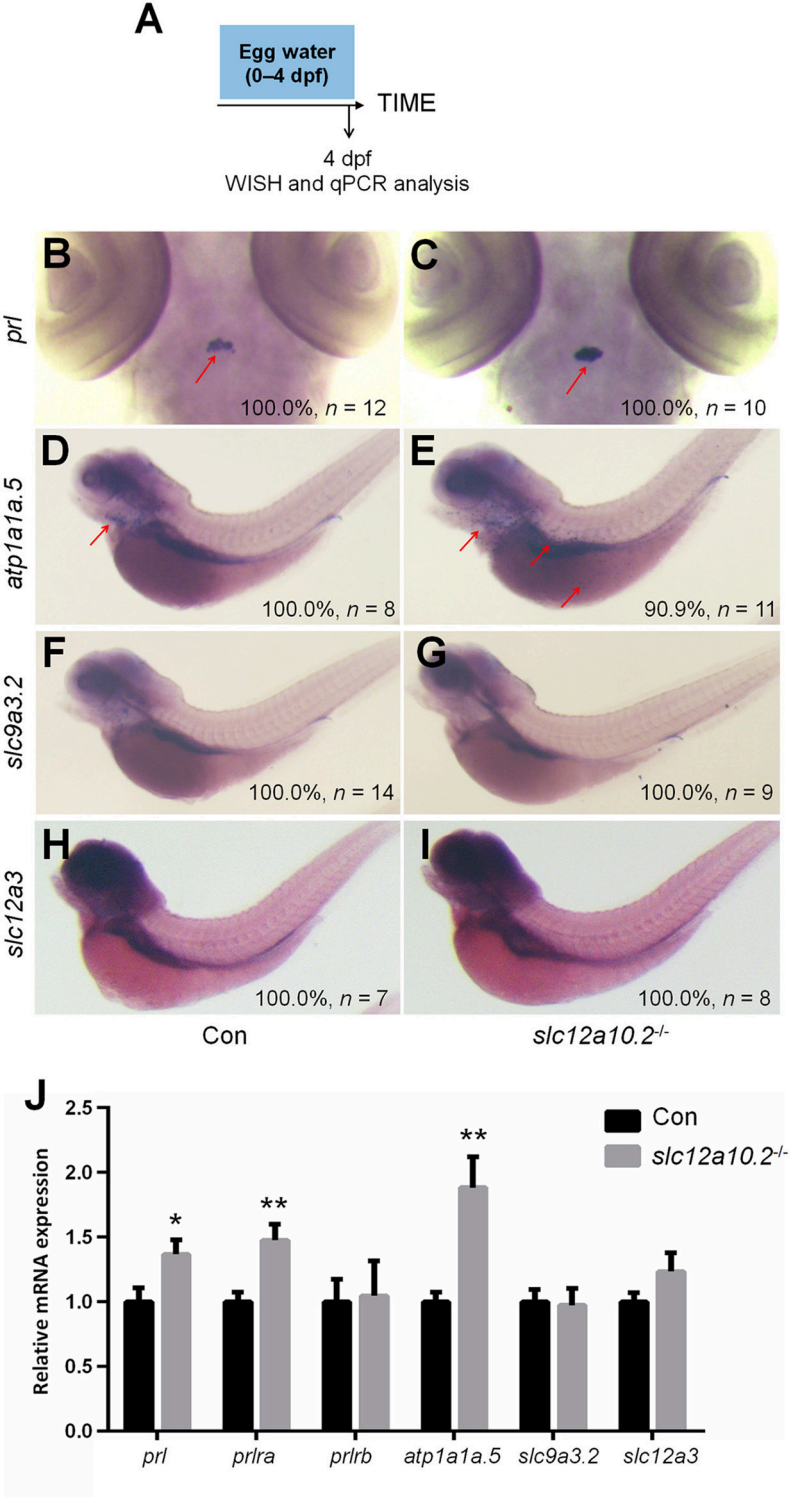
Expressions of *prl* and PRL targets in control and *slc12a10.2*-deficient larvae at 4 dpf. **(A)** Schematic diagram of the rearing timeline. Larvae were harvested at 4 dpf for WISH and qPCR analysis. **(B,C)** WISH analysis using the probe of *prl* in control and *slc12a10.2*-deficient larvae at 4 dpf. **(D,E)** WISH analysis using the probe of *atp1a1a.5* in control and *slc12a10.2*-deficient larvae at 4 dpf. **(F,G)** WISH analysis using the probe of *slc9a3.2* in control and *slc12a10.2*-deficient larvae at 4 dpf. **(H,I)** WISH analysis using the probe of *slc12a3* in control and *slc12a10.2*-deficient larvae at 4 dpf. Red arrows indicate the expressions of the nominated genes. **(J)** Expression levels of *prl, prlra, prlrb, atp1a1a.5, slc9a3.2*, and *slc12a3* in control larvae and *slc12a10.2*-deficient larvae at 4 dpf were examined with qPCR. ^*^P < 0.05. ^**^P < 0.01.

In order to better elucidate the changes in the PRL network in osmoregulation when Slc12a10.2 is absent, the expressions of downstream PRL targets were examined in control and *slc12a10.2*-deficient larvae at 4 dpf (Figure 3A). In *slc12a10.2*-deficient fish, the upregulation of pituitary *prl* and gills/skin *atp1a1a.5* was observed (Figures 3B–E). Intriguingly, the expressions of the PRL transcriptional targets, *atp1a1a.5, slc9a3.2*, and *slc12a3*, were unaffected in the pronephric duct in *slc12a10.2*-deficient fish (Figures 3D–I). These results demonstrate that *atp1a1a.5* in the gills most likely compensates for Na^+^ uptake in *slc12a10.2*-deficient fish rather than the other Na^+^ transporters in the pronephric duct. The upregulated gene expression of *atp1a1a.5* was also confirmed with qPCR (Figure 3J). It is also worth noting that *prl* and *prlra* were upregulated in *slc12a10.2*-deficient zebrafish compared to control fish (Figure 3J). In light of the previous studies reporting that *prlra* is the downstream target of PRL (5, 23, 42), this data suggests that PRL signaling is amplified in the *slc12a10.2*-deficient zebrafish.

**Figure 4:**
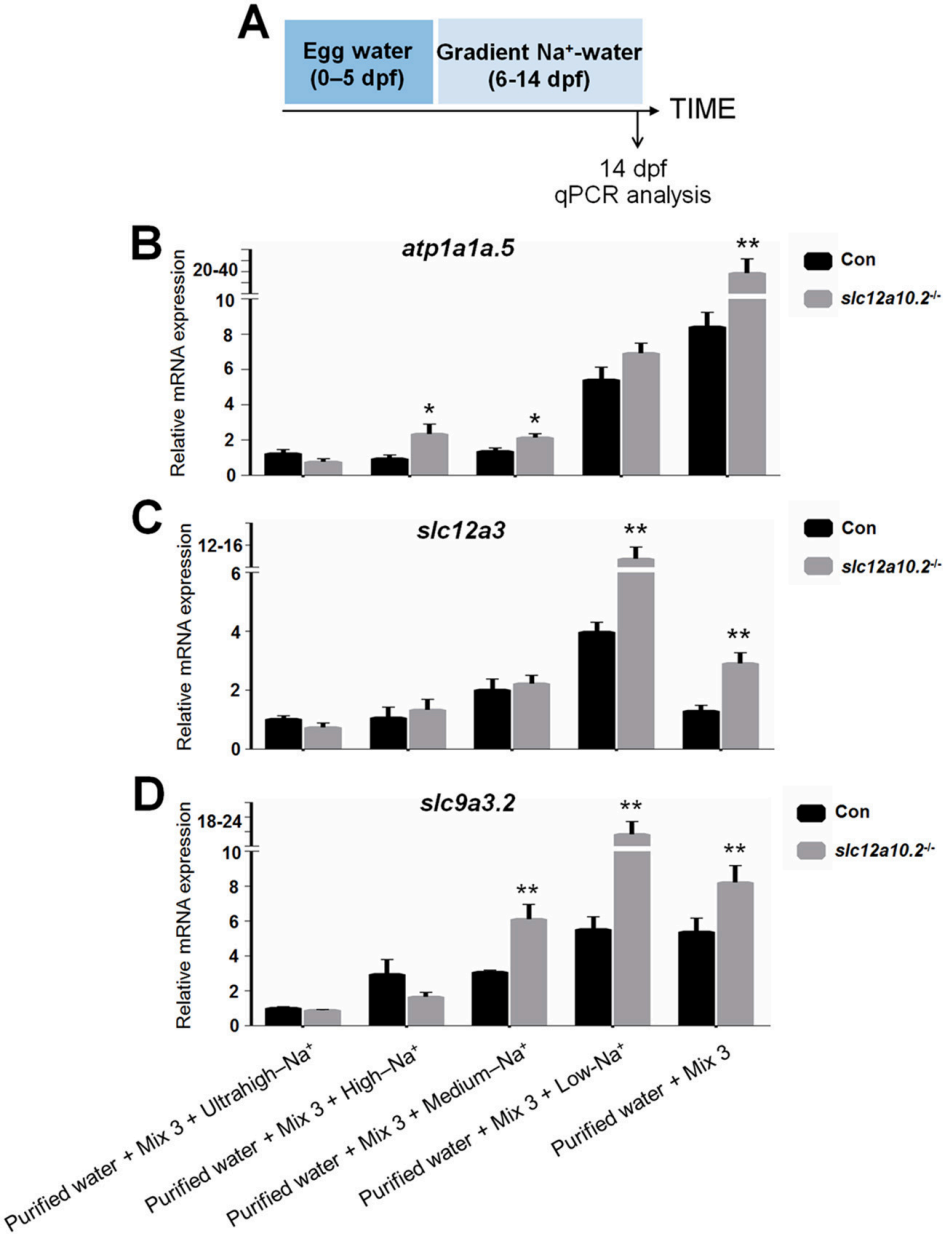
Hypo-Na^+^, not standard conditions, upregulates the expressions of the transporters. **(A)** Schematic diagram of the rearing timeline. Larvae were harvested at 14 dpf for qPCR analysis. **(B–D)** Expression levels of *atp1a1a.5, slc12a3*, and *slc9a3.2* were examined in the control and *slc12a10.2*-deficient larvae reared in purified water + Mix 3 + ultrahigh-Na^+^, purified water + Mix 3 + high-Na^+^, purified water + Mix 3 + medium-Na^+^, purified water + Mix 3 + low-Na^+^, and purified water + Mix 3, from 6 to 14 dpf. ^*^P < 0.05. ^**^P < 0.01.

We subsequently performed qPCR to examine the mRNA expressions of Na^+^ uptake-related transporters, i.e., *atp1a1a.5, slc12a3*, and *slc9a3.2*, in the *slc12a10.2*-deficient fish when reared in artificial medium with different Na^+^ concentrations (ultrahigh-, high-, medium-, low-, and 0-Na^+^ concentration) from 6 to 14 dpf. In control fish reared in the artificial medium with ultrahigh- or high-Na^+^ content, the expressions of PRL transcriptional targets, *atp1a1a.5, slc12a3*, and *slc9a3.2*, were comparable with that in the *slc12a10.2*-deficient fish. However, these genes are significantly upregulated in *slc12a10.2*-deficient fish reared in the artificial medium with low- or 0-Na^+^ content relative to control fish (Figures 4A–D). These results suggest that, though pituitary *prl* was upregulated in the *slc12a10.2*-deficient fish when reared under standard conditions, the expressions of the PRL downstream targets in the pronephric duct were not similarly upregulated. However, when the fish inhabit a Na^+^-poor environment, the PRL downstream targets, i.e., the Na^+^ transporters in pronephric duct, would be upregulated to relieve the stress of Na^+^ absorption in the gills.

### Cas9-mediated knockout of *slc12a3*

**Figure 5:**
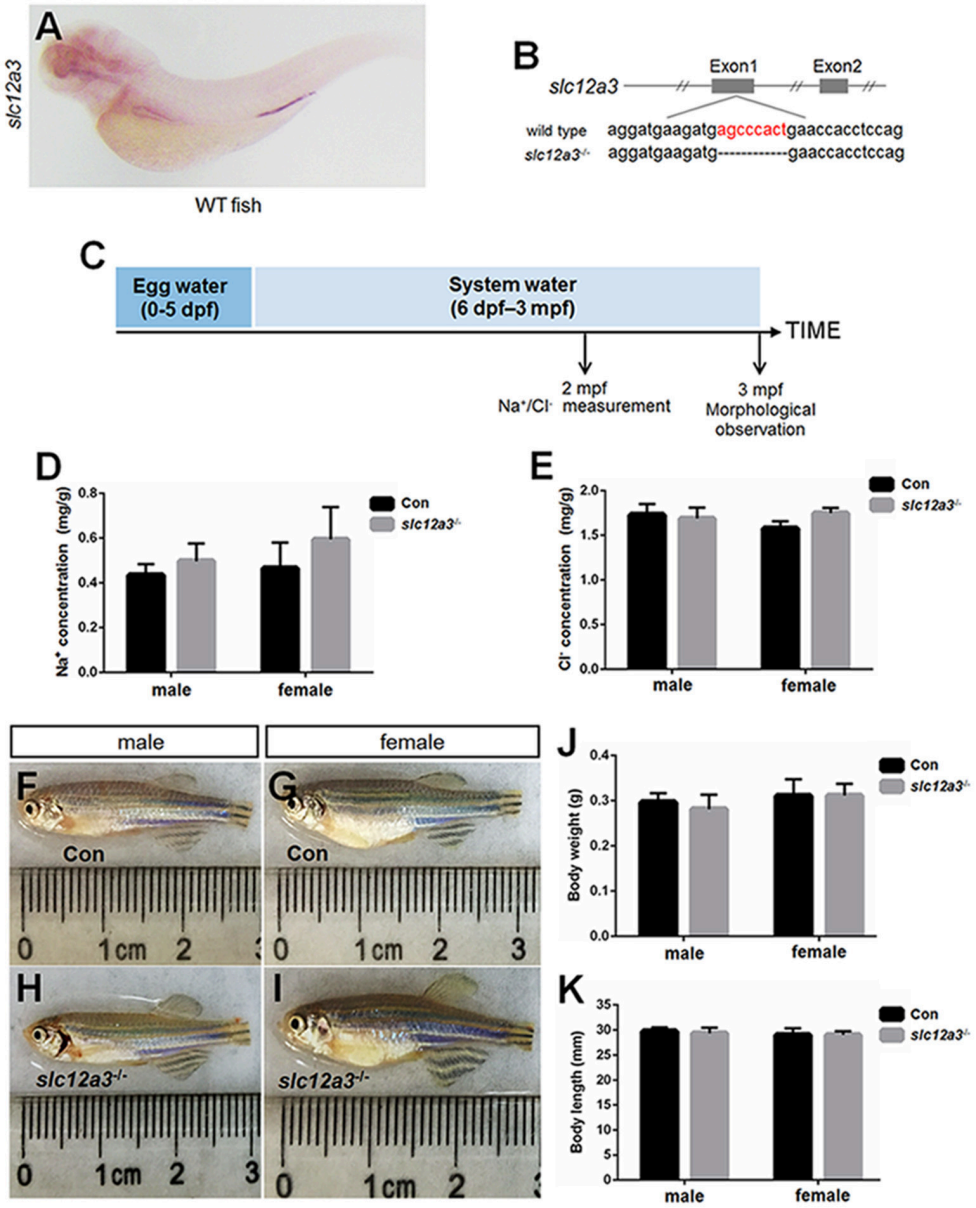
Generation of *slc12a3*-deficient zebrafish. **(A)** WISH analysis using the *slc12a3* probe in wild-type larvae at 4 dpf. **(B)** Targeted depletion of the *slc12a3* gene. The CRISPR/Cas9 target site is located at exon 1. A genotype with an 8-bp deletion was used to establish the *slc12a3* knockout line (highlighted in red). **(C)** Schematic diagram of the rearing timeline. Larvae were reared in egg water from 0 to 5 dpf and in system water after 5 dpf. The fish at 2 mpf were harvested for Na^+^ and Cl^−^ measurements. The fish at 3 mpf were harvested for morphological observation. **(D,E)** Na^+^ and Cl^−^ concentrations were measured in control and *slc12a3*-deficient male and female fish at 2 mpf, respectively. **(F–I)** The general morphological observations of control and slc12a3-deficient male and female at 3 mpf, respectively. **(F,G)** Male and female control fish at 3 mpf. **(H,I)** Male and female *slc12a3*-deficient fish at 3 mpf. **(J,K)** Body weight and body length of control and *slc12a3*-deficient male and female at 3 mpf.

WISH staining using a probe against *slc12a3* demonstrated that *slc12a3* is expressed in the pronephric duct (Figure 5A). A mutant line with an 8 bp deletion in the first exon of *slc12a3* was obtained (Figure 5B). To assess the effects of *slc12a3* on the Na^+^ and Cl^−^ concentrations in the entire zebrafish body, we measured the Na^+^ and Cl^−^ concentrations in *slc12a3*-deficient fish at 2 months post-fertilization (mpf) (Figure 5C). Surprisingly, the concentrations of Na^+^ and Cl^−^ were unaffected in the *slc12a3*-deficient zebrafish (Figures 5D,E). We also found that both male and female *slc12a3*-deficient fish were capable of surviving to adulthood in a freshwater habitat without any obvious defect (Figures 5F–I) or any significant difference in body weight and body length compared to their control siblings (Figures 5J,K).

### The *slc12a3*-depletion upregulates *PRL* and PRL transcriptional targets in the pronephric duct

**Figure 6:**
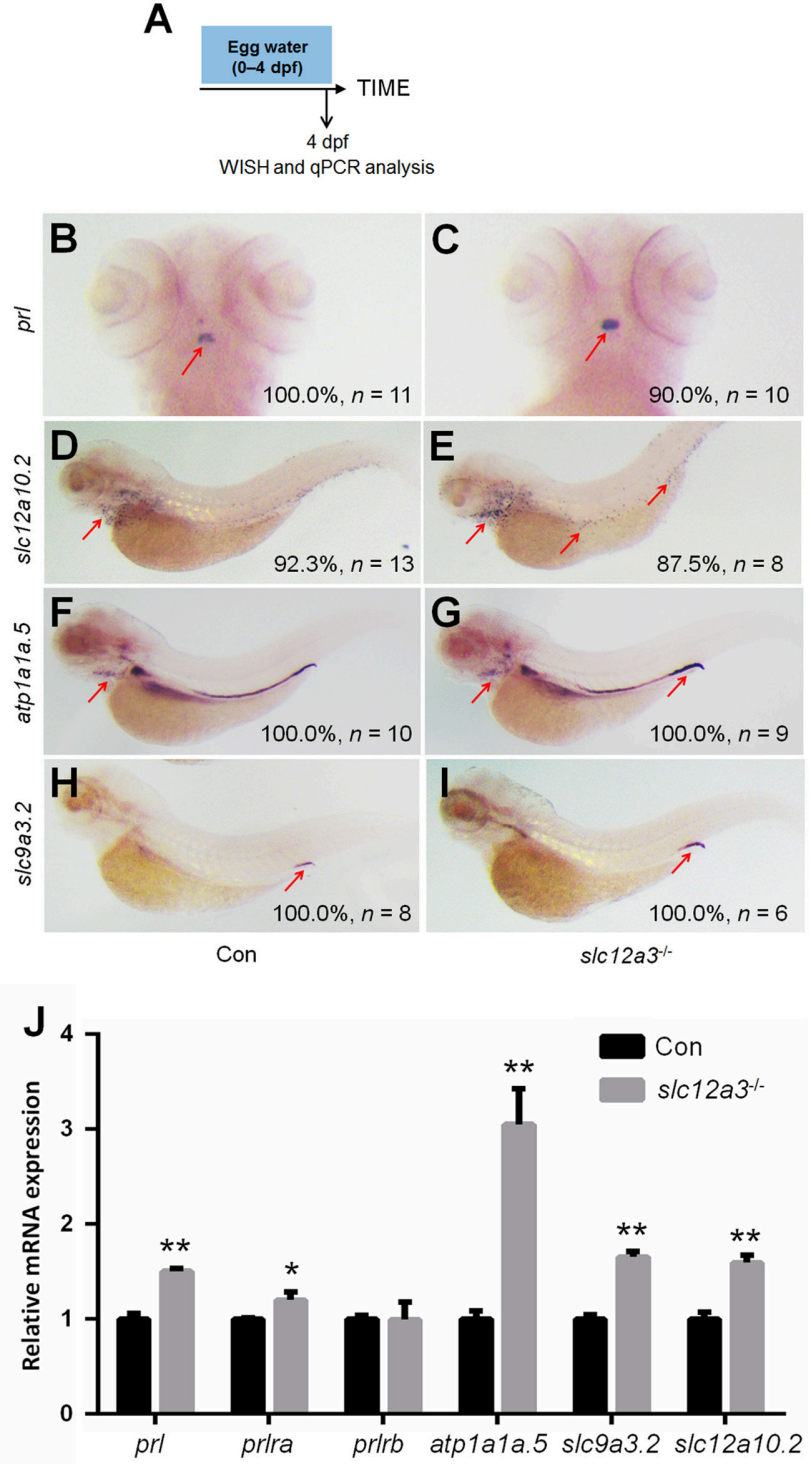
Expression patterns of *prl* and PRL targets in the *slc12a3*-deficient larvae at 4 dpf. **(A)** Schematic diagram of the rearing timeline. Larvae were harvested at 4 dpf for WISH analysis. **(B,C)** WISH analysis using the probe of *prl* in control and *slc12a3*-deficient larvae at 4 dpf. **(D,E)** WISH analysis using the probe of *slc12a10.2* in control and *slc12a3*-deficient larvae at 4 dpf. **(F,G)** WISH analysis using the probe of *atp1a1a.5* in control and *slc12a3*-deficient larvae at 4 dpf. **(H,I)** WISH analysis using the probe of *slc9a3.2* in control and *slc12a3*-deficient larvae at 4 dpf. Red arrows indicate the expressions of the nominated genes. **(J)** Expression levels of *prl, prlra, prlrb, atp1a1a.5, slc9a3.2*, and *slc12a10.2* in control larvae and *slc12a3*-deficient larvae at 4 dpf were examined with qPCR assay. ^*^P < 0.05. ^**^P < 0.01.

In *slc12a3*-deficient fish at 4 dpf, the upregulation of *prl* in the pituitary, as well as, *atp1a1a.5* and *slc12a10.2* in the gills/skin were observed (Figures 6A–G), which are partly similar to the observations in the *slc12a10.2*-deficient larvae (Figures 3B–E). We observed upregulated expressions of downstream PRL targets, including *slc9a3.2* and *atp1a1a.5*, in pronephric duct (Figures 6F–I). The upregulated expressions of *slc12a10.2, atp1a1a.5*, and *slc9a3.2* were also confirmed by qPCR analysis (Figure 6J). The expressions of *prl* and *prlra* were also significantly upregulated in *slc12a3*-deficient zebrafish compared to control fish, again reflecting the fact that PRL is increased in *slc12a3*-deficient fish compared with control fish (Figure 6J). The overall response of the downstream PRL targets, both in the gills/skin and pronephric duct, to *slc12a3*-deficiency are quite different with the observations in *slc12a10.2*-deficient larvae (Figure 3).

## Discussion

In mammals, NCC (encoded by *slc12a3*), a thiazide-sensitive membrane protein, is a critical transporter promoting salt reabsorption in the apical membranes of the mammalian distal convoluted tubules of the pronephric duct (43). In zebrafish, two members of the *slc12a* family, *slc12a10.2* (encodes NCC-2b or NCC-like) and *slc12a3* (encodes NCC), expressed in the gills/skin and pronephric duct, respectively, are both identified as PRL transcriptional targets (17, 35, 36). In the present study, models with increased endogenous PRL signaling were generated by the knockout of *slc12a10.2* and *slc12a3*. By investigating the functional roles of *slc12a10.2* and *slc12a3* in zebrafish, the novel regulatory network of PRL signaling was elucidated. Specifically, we demonstrated differential expression of downstream targets in the gills and pronephric duct in zebrafish deficient in *slc12a10.2* or *slc12a3*.

**Figure 7:**
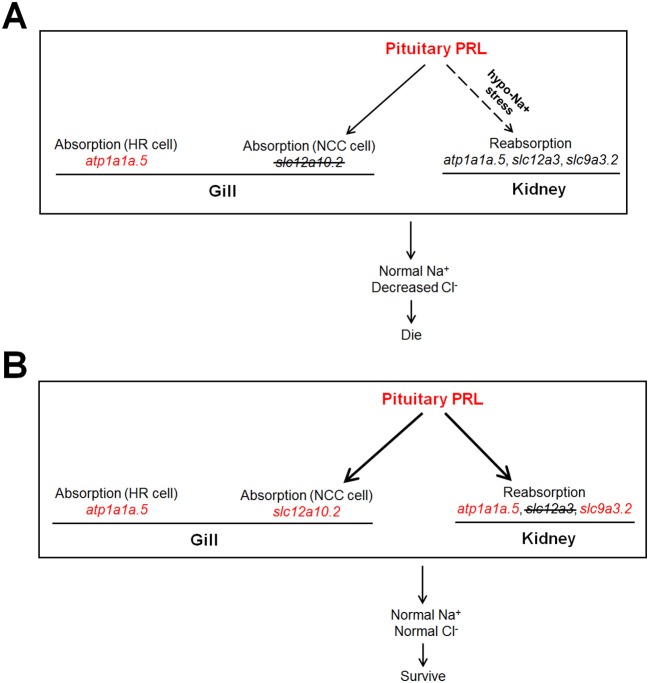
The dissected regulatory network of PRL signaling in the *slc12a10.2*- and *slc12a3*-deficient fish, respectively. **(A,B)** The generations of the model with increased endogenous PRL in zebrafish. Either depletion of *slc12a10.2* or *slc12a3* could result in increased endogenous PRL effectively. **(A)** Although pituitary *prl* is upregulated in the *slc12a10.2*-deficient fish, the responses of PRL downstream targets in pronephric duct to upregulated *prl* is not observed, as this pathway could only be conditionally activated under the hypo-Na^+^ stress. The Na^+^ absorption maintenance by upregulated *atp1a1a.5* in the gills/skin of the *slc12a10.2*-deficient larvae is evidently observed, which probably compensate to maintain the systemic Na^+^ concentration. However, the PRL downstream targets in the pronephric duct failed to respond to the increased pituitary PRL. **(B)** The overall responses of Na^+^ and Cl^−^ absorption both in the gills/skin and reabsorption in the pronephric duct compensate to maintain normal levels of Na^+^ and Cl^−^ in the *slc12a3*-deficient fish. The upregulated expressions of the nominated genes are highlighted in red.

In the present study, the increased expression of pituitary *prl* was observed in both the *slc12a10.2*- and *slc12a3*-deficient zebrafish, supporting the notion that PRL is the key mediator in maintaining the expressions of *slc12a10.2* and *slc12a3*. This study also provides new insight that suggests that PRL is susceptible to deficiencies in downstream ion transporters, as evidenced by the increased transcriptional levels of *prl* from WISH and qPCR analysis (Figures 3, 6). It has been reported that intraperitoneal injection of ovine PRL increased *prlra* transcript (5). Intriguingly, the upregulation of *prlra* was observed in both of the knockout lines, indicating that PRL is increased when the downstream ion transporter was depleted (Figures 3, 6, 7). Although the protein levels of PRL were not examined in the present study, the amplified PRL signaling was still determined in the two knockout lines.

No significant difference in systemic Na^+^ levels was observed in *slc12a10.2*- or *slc12a3*-deficient zebrafish. From the results of WISH, we identified other underlying compensators for Na^+^ uptake, i.e., upregulated *atp1a1a.5* and *atp1a1a.5*/*slc12a10.2* in the gills/skin of the *slc12a10.2*-deficient fish and *slc12a3*-deficient fish, respectively. The expressions of PRL transcriptional targets to increased levels of pituitary PRL in the gills/skin and pronephric duct were observed in the *slc12a3*-deficient zebrafish: elevated expressions of *slc9a3.2* (in the pronephric duct), *slc12a10.2* (in the gills/skin), and *atp1a1a.5* (both in the gills and pronephric duct) were simultaneously observed in *slc12a3*-deficient fish (Figure 6). Interestingly, the expressions of PRL targets in response to elevated levels of endogenous PRL in the pronephric duct observed in *slc12a3*-deficient fish were not seen in *slc12a10.2*-deficient fish. It is clear that the regulation of systemic salt uptake are more complex than previously anticipated, as the absorption of both Na^+^ and Cl^−^ take place in the pronephric duct and gills/skin, and these two organs work cooperatively to regulate the systemic levels of Na^+^ and Cl^−^ (9, 44). It has been reported that *slc12a10.2* is the transcriptional target of PRL, as both knockdown and knockout of *prl* result in the downregulation of *slc12a10.2* in the gills/skin of zebrafish (10, 17). It has been shown that the ovine PRL supplement is sufficient to upregulate *slc12a10.2* expression both *in vivo* and *in vitro*. The upregulation of *slc12a10.2* by ovine PRL indicates that *slc12a10.2* can be upregulated by exogenous PRL supplementation (5). These previous results are in agreement with the observed upregulation of *slc12a10.2* in response to elevated levels of endogenous PRL in the gills/skin of *slc12a3*-deficient fish (Figure 6).

Unlike the gill *slc12a10.2* and pronephric *slc9a3.2*, which are competent to respond to elevated levels of endogenous PRL caused by *slc12a3* depletion, the pronephric duct *slc12a3* did not respond to elevated levels of PRL caused by the depletion of *slc12a10.2* (Figures 3, 6). It seems that the pronephric duct transporters, *slc12a3, slc9a3.2*, and *atp1a1a.5*, undergo differential transcriptional regulation of PRL signaling in the gills/skin of *slc12a10.2*-deficient zebrafish. This, therefore, leads to the failed response of the pronephric duct genes *slc12a3* and *slc9a3.2* to the increased levels of pituitary PRL in the *slc12a10.2*-deficient fish when reared under the standard conditions (Figures 3F–I). The differential response of the gills/skin and pronephric duct to manipulated PRL signaling is in agreement with the previous study. Although the expressions of *prl, slc12a10.2*, and *slc9a3.2*, as well as, the plasma levels of PRL, increase in response to a low salinity environment (18, 45–47), exogenous supplementation with ovine PRL, which is sufficient to upregulate *slc12a10.2*, failed to upregulate *slc9a3.2* (5). Taken together, in regard to the *slc12a10.2*-deficiency, we conclude that when fish are reared under standard conditions with suitable levels of Na^+^, the pronephric duct transporters do not respond to the elevated pituitary PRL caused by the depletion of *slc12a10.2* (Figures 3F–I). However, *slc12a10.2*-deficient zebrafish need the intact response in the gills/skin and pronephric duct to cope with hypo-Na^+^ stress. Thus, this PRL pathway potentially helps zebrafish rapidly acclimate to dramatic reductions in Na^+^ concentrations and adapt to natural conditions (Figures 4A–D).

Cl^−^ is the main anion with biological functions, such as cell volume regulation, transport of salt and fluid across epithelia, muscle contraction, charge compensation, and acidification of intracellular organelles (48). In humans, mutation of the chloride transporters, *clc-1* or *clc-kb*, causes myotonia and Bartter's syndrome, respectively. In mice, knockout of *clc-k1* leads to diabetes insipidus (49, 50), whereas the *clc-2* knockout mice demonstrated blindness, sterility, and leukodystrophy by unknown reasons (51–53). More specifically, knockout of the chloride transporter *clcc1* in zebrafish leads to damaged retinal morphology and function and lethality at 11 dpf (54). It has been shown that both *slc12a10.2* and *slc12a3* are responsible for Cl^−^ uptake, and gill mRNA expression of *slc12a10.2* was induced by a low-Cl^−^ environment; however, a normal Cl^−^ concentration in *slc12a3*-deficient fish at 2 mpf was observed, which probably resulted from the upregulation of *slc12a10.2* in the gills/skin. We also demonstrated that *slc12a10.2*-deficiency in fish was lethal due to defective Cl^−^ uptake. This result clearly demonstrated that Slc12a10.2 plays an essential role in Cl^−^ uptake; however, the role Slc12a3 plays in Cl^−^ absorption could not be completely concluded, as the potential compensatory effect of Slc12a10.2 may contribute to chloride absorption.

The naturally occurring mutations found in the human *SLC12A3* gene have been identified in patients suffering from a salt-wasting disorder called Gitelman's syndrome (55). It is interesting that, unlike the mammals, zebrafish possess multiple subtypes of NCC encoded by distinct mRNAs (36). In larval fish, the relative abundance of *slc12a10.2* and *slc12a3* differ during early embryonic development (Figures 1, 5). Although downregulation of *slc12a10.2* and *slc12a3* were observed in *prl*-knockdown or *prl*-knockout fish (10, 17), distinct expression patterns suggest that they may undergo a complicated regulatory network of PRL signaling. The phenotypes associated with the *SLC12A3* gene mutation is in sharp contrast to our observations of the *slc12a10.2*- and *slc12a3*-deficient fish. This suggests that overlapping roles of Slc12a10.2 and Slc12a3 in maintaining Na^+^/Cl^−^ absorption may exist in zebrafish. However, the different phenotypes of *slc12a10.2*- and *slc12a3*-deficient fish also suggest that Slc12a10.2 and Slc12a3 exhibit diversified functions in maintaining Na^+^/Cl^−^ absorption. The different results obtained from the *slc12a10.2*- and *slc12a3*-deficient fish, e.g., the conditional and non-conditional-activated PRL signaling, may be caused by the diversified functions of the two transporters in maintaining Na^+^/Cl^−^ absorption. Such differences between the two transporters in osmoregulation in teleosts were also observed in a tilapia PRL study, where tilapia PRL_188_ injection resulted in a dose-dependent effect on ion retention, as evidenced by an increase in the plasma levels of Na^+^ and Cl^−^, but PRL_177_ injection caused only slight increases in plasma Na^+^ and Cl^−^ that were not dose-dependent (56).

In the present study, a zebrafish model was used for the dissection of the PRL regulatory signaling network, as zebrafish inhabit hypotonic freshwater environments and have emerged as an important model for investigating osmoregulation, especially with regard to the genetic editing and manipulation. To our knowledge, there has been no report of a knockout model with increased endogenous PRL signaling or the evaluation of responsive expressions of ion transporters in zebrafish. Unlike the previous studies established from the knockout or knockdown experiments of pituitary *prl*, the present study regarding the knockout of *slc12a10.2* and *slc12a3* in zebrafish are more integrative to the dissection of the regulatory network of PRL signaling by exploring the transcriptional expressions of PRL targets in response to increase in pituitary PRL caused by the depletion of ion transporters that are tissue-specific (Figure 7). To further our understanding of the physiological roles of NCC transporters, it would be useful if future studies obtain the *slc12a10.2*/*slc12a3* double-knockout fish. Moreover, the detailed mechanisms concerning the failed responses of the transporters in the pronephric duct to elevated levels of pituitary PRL in the *slc12a10.2*-deficient fish require further research. Thus, it would be a challenging, but important issue to investigate whether there is any other hypo-Na^+^-mediated transcriptional factor that may be responsible for the conditionally-activated transcription of the transporters in the pronephric duct of *slc12a10.2*-deficient fish (Figure 7).

## Author contributions

TS and YS conducted most of the experiments for this work. YG, XJ, and JH provided help with genotyping and fish rearing. GZ performed training and provided insights for this work. GZ wrote the paper and prepared all of the figures. ZY initiated and supervised the research team and revised the paper. All the authors approved the final manuscript.

### Conflict of interest statement

The authors declare that the research was conducted in the absence of any commercial or financial relationships that could be construed as a potential conflict of interest.
